# Insulin sensitivity in long-lived growth hormone-releasing hormone knockout mice

**DOI:** 10.18632/aging.103588

**Published:** 2020-07-08

**Authors:** Fang Zhang, Mert Icyuz, Zhenghui Liu, Michael Fitch, Liou Y. Sun

**Affiliations:** 1 Department of Biology, University of Alabama at Birmingham, Birmingham, AL 35254, USA; 2 Department of Obstetrics and Gynecology, University of Colorado, Anschutz Medical Campus, Aurora, CO 80045, USA

**Keywords:** GHRH^-/-^ mice, tissues-specific insulin signaling, hyperinsulinemic-euglycemic clamp, AKT/S6, ERK1/2

## Abstract

Our previous studies showed that loss-of-function mutation of growth hormone releasing hormone (GHRH) results in increased longevity and enhanced insulin sensitivity in mice. However, the details of improved insulin action and tissue-specific insulin signaling are largely unknown in this healthy-aging mouse model. We conducted hyperinsulinemic-euglycemic clamp to investigate mechanisms underlying enhanced insulin sensitivity in growth hormone (GH) deficient mice. Further, we assessed *in vivo* tissue-specific insulin activity via activation of PI3K-AKT and MAPK-ERK1/2 cascades using western blot. Clamp results showed that the glucose infusion rate required for maintaining euglycemia was much higher in GHRH^-/-^ mice compared to WT controls. Insulin-mediated glucose production was largely suppressed, whereas glucose uptake in skeletal muscle and brown adipose tissue were significant enhanced in GHRH^-/-^ mice compared to WT controls. Enhanced capacity of insulin-induced activation of the PI3K-AKT and MAPK-ERK1/2 signaling were observed in a tissue-specific manner in GHRH^-/-^ mice. Enhanced systemic insulin sensitivity in long-lived GHRH^-/-^ mice is associated with differential activation of insulin signaling cascades among various organs. Improved action of insulin in the insulin sensitive tissues is likely to mediate the prolonged longevity and healthy-aging effects of GH deficiency in mice.

## INTRODUCTION

Impaired growth hormone (GH) signaling is associated with human familial longevity [1], and also with remarkable extended longevity in mouse models [2], including Ames (*Prop-1^df^*) and Snell (*Pit-1^dw^*) dwarf mice [3, 4], GH receptor/binding protein-deficient (GHRKO) mouse [5], *Ghrhr^lit/lit^* mutation mouse [6] and GH-releasing hormone knockout (*ghrh^-/-^*) mouse [7]. These GH signaling-impaired mice with improved longevity have decreased circulating insulin levels and enhanced systemic insulin sensitivity [8]. However, the details of improved insulin action and tissue-specific insulin signaling are largely unknown in the context of GH deficient mice.

Insulin acts as the central hormone controlling the carbohydrate, lipid and protein metabolism. In the liver, insulin stimulates glucose uptake [9], also promotes glycogen synthesis by inhibiting the activation of glucose-6-phosphatase and phosphorylation of glycogen synthase and by suppressing glucose production and the rate of glycogen breakdown [10]. Excessive insulin signaling promotes *de novo* lipid synthesis in hepatocytes [11]. Muscle tissue is another major target of insulin signaling. The effects of insulin on muscle tissue include promotion of glucose uptake and activation of hexokinase and 6-phosphofructokinase to increase glycolysis [12]. In addition, insulin promotes muscular glycogen synthesis and inhibits glycogen breakdown [13]. In white adipose tissue (WAT), insulin increases glucose uptake and glycolysis by activating glucose transport in adipocytes [14]. Moreover, insulin inhibits lipolysis via enhancing lipid storage and decreasing circulating fatty acid levels [14].

Insulin regulates mineral transport and gluconeogenesis in the kidney [15] and induces activation of β-catenin in lung tissue and airway smooth muscle cells, which is causally related to the development of asthma-like phenotypes in the lung [16]. Insulin signaling influences multiple functions in the heart, including metabolic substrate preference, cell size, and the response of the heart to ischemia and hypertrophy [17]. Insulin plays critical roles in synaptogenesis and synaptic remodeling in the central nervous system [18]. Importantly, insulin modulates glucose utilization in the brain [19] and facilitates learning, cognition and motivation at optimal levels [20].

At molecular level, insulin exerts its action through insulin-mediated autophosphorylation of receptor tyrosine kinase [21]. Insulin signaling is initiated via activation of receptors and triggers a cascade of phosphorylation events in two major signaling pathways [22]. In the metabolic pathway, insulin-activated insulin receptor substrate recruits and activates PI3K [23]. Activation of PI3K promotes generation of PIP3, which recruits PDK1 to phosphorylate AKT at threonine 308 and mTORC2 phosphorylates AKT at serine 473 [24–26]. Subsequently, activated AKT phosphorylates mTORC1, which in turn activates Ribosomal protein S6 (S6) to promote protein synthesis. Further, insulin-induced phosphorylation of AKT decrease activation of GSK3 resulting in reduced phosphorylation of glycogen synthase and increased fractional activity of glycogen synthase, which leads to increase in glycogen synthesis in skeletal muscle [27–29]. In the mitogenic pathway, insulin-induced Grb2-SOS-Ras signaling leads to activation of ERK [30].

GHRH^-/-^ mice with isolated GH deficiency were characterized by dwarfism, enhanced insulin sensitivity and extended longevity (more than 40 % in both sexes) [7]. Importantly, compared with control, GHRH^-/-^ mice and other GH-impaired mice with long-lived have altered activation of insulin-mediated AKT or/and ERK1/2 signaling pathway, which may play a role in their extended longevity [7, 26]. Insulin plays global roles in whole body, however, the details of improved insulin action, particularly tissue-specific insulin signaling are largely unknown in this healthy-aging mouse model. Thus, we employed hyperinsulinemic-euglycemic clamp to measure whole-body insulin sensitivity and immunoblotting to examine the two major insulin signaling pathways; PI3K/AKT/S6 and MAPK/ERK in various tissues.

Our study shows that enhanced systemic insulin sensitivity in long-lived GHRH^-/-^ mice is associated with differential activation of insulin signaling cascades among various organs. This improved insulin action in the target tissues is likely to mediate the prolonged longevity and healthy-aging effects of GH deficiency in mice.

## RESULTS

### Physiological parameters in GHRH deficient mice

**Figure 1:**
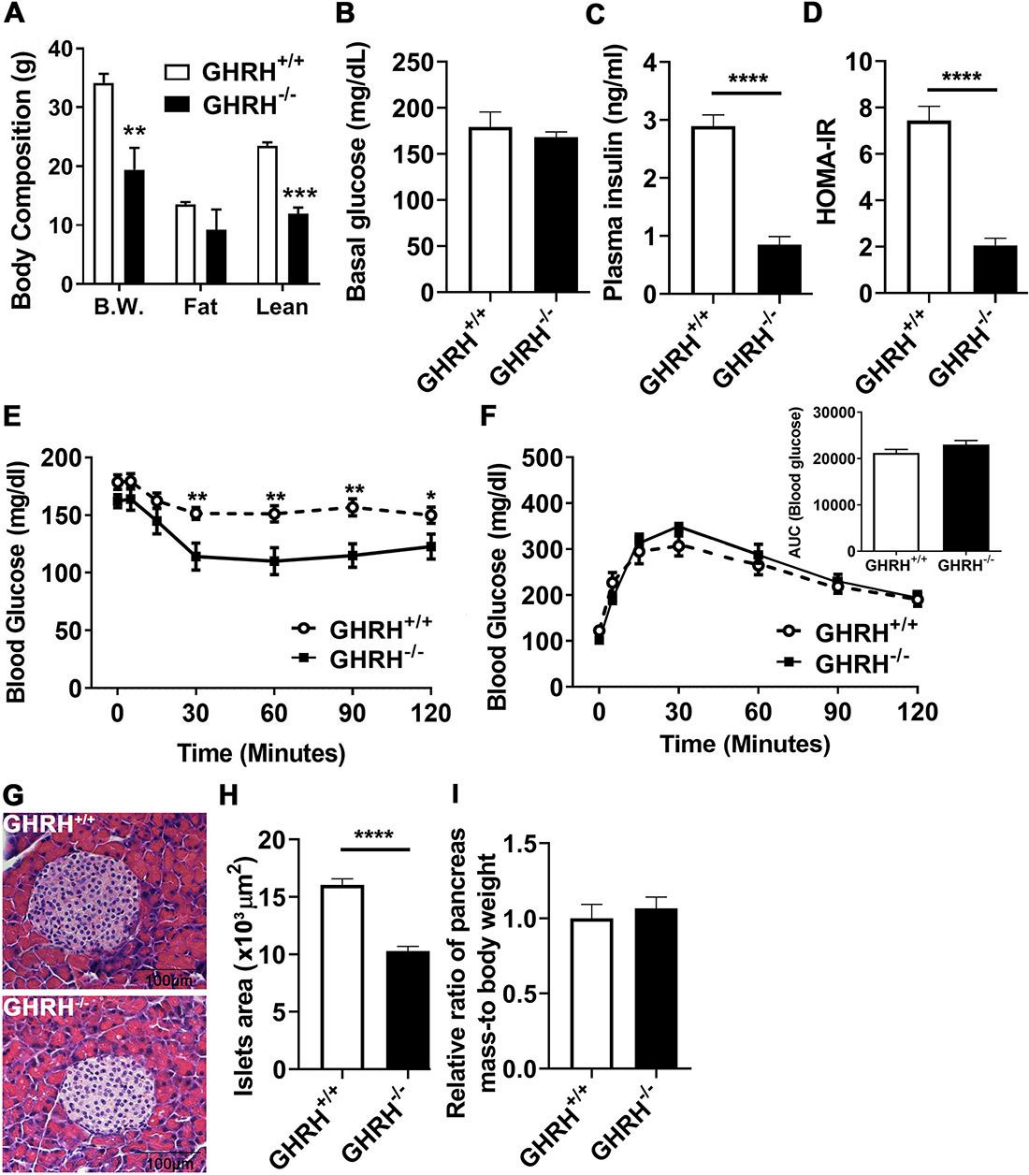
**Glucose homeostasis and insulin sensitivity in GHRH^-/-^ mice.** (**A**) Body composition. (**B**–**D**) 4-hour fasted glucose, plasma insulin and homeostatic model for assessment of insulin resistance (HOMA-IR). (**E**) Intraperitoneal insulin tolerance test (IPITT). 4 hours fasted mice were i.p. injected with porcine insulin (1 IU/kg of body weight). (**F**) Intraperitoneal glucose tolerance test (IPGTT) and area under the curve analysis (AUC). 16 hours fasted mice were i.p. injected with glucose (1 g/ kg of body weight). (**G**) Representative photomicrographs of pancreatic islet sections stained with H&E staining. Scale bar is 100 μm. (**H**) Average of pancreatic islets. Total 50 pancreatic islets from 5 mice for each genotype were quantitated. (**I**) Relative ratio of pancreas mass (g) versus whole body weight (g). Data (means ± sem) was expressed as fold change compared to WT controls (defined as 1.0). All data were represented as the means ± sem (n=14 in each genotype for IPITT and IPGTT; n=8 in each group for fasted glucose, plasma insulin and HOMA-IR; n=5 in each genotype for evaluation of average pancreatic islets and relative ratio of pancreas mass). Statistical analysis was performed by unpaired Student’s t-test, * *P* < 0.05, ** *P* < 0.01 and **** *P* < 0.0001.

Consistent with our previous observations, the body weight of GHRH^-/-^ mice is significantly lower than WT controls (Figure 1A). Dual-energy X-ray absorptiometry revealed that GHRH^-/-^ mice have significantly decreased lean mass, while showing no significant difference in fat mass between GHRH^-/-^ mice and WT controls (Figure 1A). Body weight-adjusted fat mass was significantly increased in GHRH^-/-^ mice compared to WT controls [31]. We did not observe difference in basal glucose levels (Figure 1B). However, plasma insulin concentration (Figure 1C) and HOMA-IR index (Figure 1D) were dramatically reduced in GHRH^-/-^ mice compared to WT controls. Compared to WT controls, GHRH^-/-^ mice have significantly enhanced insulin sensitivity (Figure 1E), despite the normal glucose tolerance results (Figure 1F). Moreover, we assessed pancreatic islet size in GHRH^-/-^ mice with hematoxylin and eosin staining. The average islet size in GHRH^−/−^ mice was decreased 66 % (*p* < 0.0001) compared to WT controls (pancreatic islet size: WT=15 x 10^3^ μm^2^, *n* = 50 versus GHRH^−/−^=10 x 10^3^ μm^2^, *n* = 50; units expressed as ×10^3^ μm^2^) (Figure 1G and 1H). To analyze correlation of insulin level and the size of pancreatic islets, we used relative ratio of pancreatic mass to body-weight as a consistent control and found that no significant difference between GHRH^-/-^ mice and WT controls (Figure 1I), thus decreased pancreatic islet size may be related to the reduced insulin level in GHRH^-/-^ mice.

### GHRH deficiency enhances insulin sensitivity in mice

**Figure 2:**
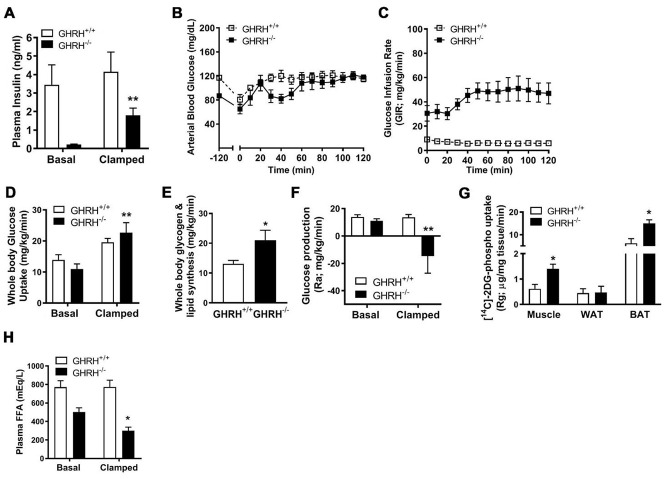
**Various metabolic parameters during hyperinsulinemic-euglycemic clamp of GHRH^-/-^ mice.** (**A**) Plasma insulin. (**B**) Blood glucose. (**C**) Glucose infusion rate. (**D**) Whole body glucose uptake. (**E**) Whole body glycogen and lipid synthesis. (**F**) Repression of rate of endogenous glucose production. (**G**) [^14^C] 2DG uptake into gastrocnemius, epididymal white and brown adipose tissue. (**H**) Plasma free fatty acids. All data were represented as mean ± sem (n = 4-5 mice for each genotype). Statistical analysis was performed by unpaired Student’s t-test, * P < 0.05 and ** P < 0.01 versus baseline time point (**D**) or versus vehicle within time points (**A** and **E**–**H**).

To gain further insight into the mechanisms underlying enhanced insulin sensitivity in GH deficiency, we conducted hyperinsulinemic-euglycemic clamps (2.5 mU/kg/min) in conscious GHRH^-/-^ mice and WT controls. Constant with our prior findings [7], fasting (basal) plasma insulin concentrations are reduced in GHRH^-/-^ mice compared with WT (Figures 1C and 2A). During the steady-state portion of the clamp (t=80-120 min), circulating insulin was significantly elevated 15-fold in GHRH^-/-^ mice (basal=0.12 ng/ml versus clamped=1.81 ng/ml), and was slightly elevated in WT mice (basal=3.3 ng/ml versus clamped=4.0 ng/ml, Figure 2A). However, GHRH^-/-^ mice were still characterized by lower insulin levels as compared to WT controls (Figure 2A). Insulin sensitivity, as defined by the glucose infusion rate (GIR) necessary to maintain euglycemia (Figure 2B), was dramatically elevated in GHRH^-/-^ mice (48 mg/kg/min) as compared to WT mice (14 mg/kg/min, Figure 2C). Insulin-stimulated whole-body glucose uptake was not significant different in WT mice compared to its basal line, however, insulin markedly increased whole-body glucose uptake in GHRH^-/-^ mice (11 mg/kg/min to 22 mg/kg/min, Figure 2D), suggesting enhanced insulin-stimulated glucose metabolism. Consistent with enhanced insulin sensitivity, insulin-stimulated whole-body glycogen/lipid synthesis was elevated in GHRH^-/-^ mice compared to WT mice (Figure 2E). Interestingly, insulin suppressed hepatic glucose production to a greater extent in the GHRH^-/-^ mice compared to WT mice (Ra, Figure 2F). In addition to the enhanced insulin action observed in hepatic tissue, we observed enhanced insulin-stimulated [^14^C]-2DG in muscle and brown adipose tissue (Rg, Figure 2G) of GHRH^-/-^ mice. While basal plasma free fatty acid (FFA) concentrations did not differ between the GHRH^-/-^ and WT mice, we found that insulin-stimulated suppression of plasma FFA was greater in GHRH^-/-^ mice compared to WT under clamped conditions (Figure 2H). Taken together, these data suggest that GH plays an important role in insulin-regulated glucose and lipid metabolism.

### Insulin-stimulated activation of AKT/S6 cascade in metabolically active tissues of GHRH^-/-^ mice

Insulin plays a major role in many metabolic actions, including glycogen deposition, stimulation of lipogenesis, inhibition of lipolysis, repression of gluconeogenesis, and regulation of glucose uptake via canonical PI3K/AKT and MAPK/ERK cascades [32]. Our IPITT data indicated that the curve of blood glucose significantly dropped in GHRH^-/-^ mice with insulin treatment in 15-30 min, compared that with WT mice (Figure 1E), thus we first sought to examine tissue-specific insulin sensitivity by activation of AKT/S6 cascade in metabolically active tissues of GHRH^-/-^ mice with tissues collected 20 min after insulin treatment.

**Figure 3:**
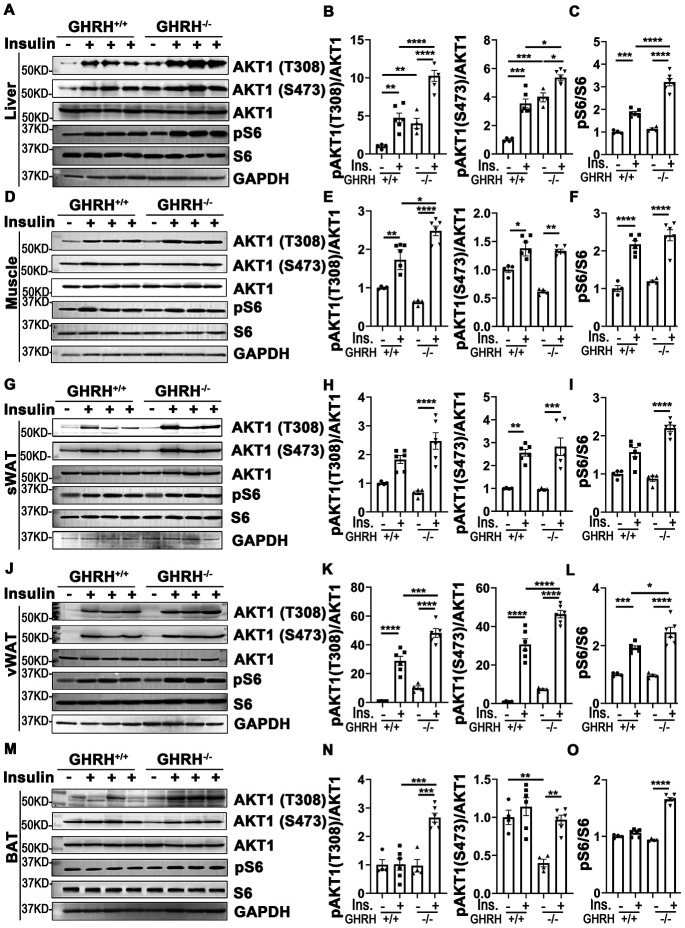
**Insulin-induced activation of AKT/S6 cascade in metabolically active tissues of GHRH^-/-^ mice.** (**A**–**C**) Activation of AKT and S6 in liver. (**D**–**F**) Activation of AKT and S6 in skeletal muscle. (**G**–**I**) Activation of AKT and S6 in subcutaneous white adipose tissue. (**J**–**L**) Activation of AKT and S6 in visceral white adipose tissue. (**M**–**O**) Activation of AKT and S6 in interscapular brown adipose tissue. The 4 hours fasted mice were injected i.p. with porcine insulin (1 IU/kg of body weight). After 20 min, tissues were collected to perform western blots. All data (means ± sem) are expressed as fold change compared to vehicle treated WT controls (defined as 1.0) (n=4 for WT group; n=6 for GHRH^-/-^ mice). Statistical analysis was performed by unpaired Student’s t-test, * *P* < 0.05, ** *P* < 0.01, *** *P* < 0.001 and **** *P* < 0.0001.

As shown in Figure 3A–3L, in both WT and GHRH^-/-^ mice, AKT1 phosphorylation on site Thr-308 was significantly induced in liver, skeletal muscle and white adipose tissues 20 minutes after intraperitoneal insulin injection compared to saline-injected control. Basal hepatic phosphorylation of AKT1 on Thr-308 was higher in GHRH^-/-^ than WT mice, while no significant difference was detected in skeletal muscle, WAT and BAT. Interestingly, the insulin-stimulated phosphorylation of AKT1 (Thr-308) was significantly higher in the liver, skeletal muscle, visceral white adipose tissue (vWAT) and BAT of GHRH^-/-^ mice compared to WT mice with insulin stimulation (Figure 3).

Insulin increases phosphorylation of AKT1/2 at Thr-308/309 and Ser-473/474 sites [33]. As shown in Figure 3, AKT1 phosphorylation on Ser-473 was significantly increased in liver, skeletal muscle and WAT in both genotypes following insulin injection. The basal and insulin-stimulated hepatic pAKT (Thr-473) was significantly higher in GHRH^-/-^ mice compared to WT controls (Figure 3A and 3B). Intriguingly, in BAT, insulin resulted in dramatically increased levels of pAKT in GHRH^-/-^ mice, but not in WT controls (Figure 3M and 3N).

We next examined the insulin-induced phosphorylation of ribosomal protein S6, which is activated by phosphorylated AKT [34]. Insulin-activated S6 was observed in liver, skeletal muscle and vWAT in both genotypes (Figure 3). Furthermore, insulin-induced pS6 was significantly higher in liver and vWAT of GHRH^-/-^ mice compared to WT mice (Figure 3A, 3C, 3J and 3L).

These data show that the liver, skeletal muscle, white adipose tissues are the most sensitive to insulin stimulation in both genotypes, while brown adipose tissue has increased insulin sensitivity in GHRH^-/-^ mice by activation of AKT/pS6 cascade.

### Activation of AKT in other tissues of GHRH^-/-^ mice

**Figure 4:**
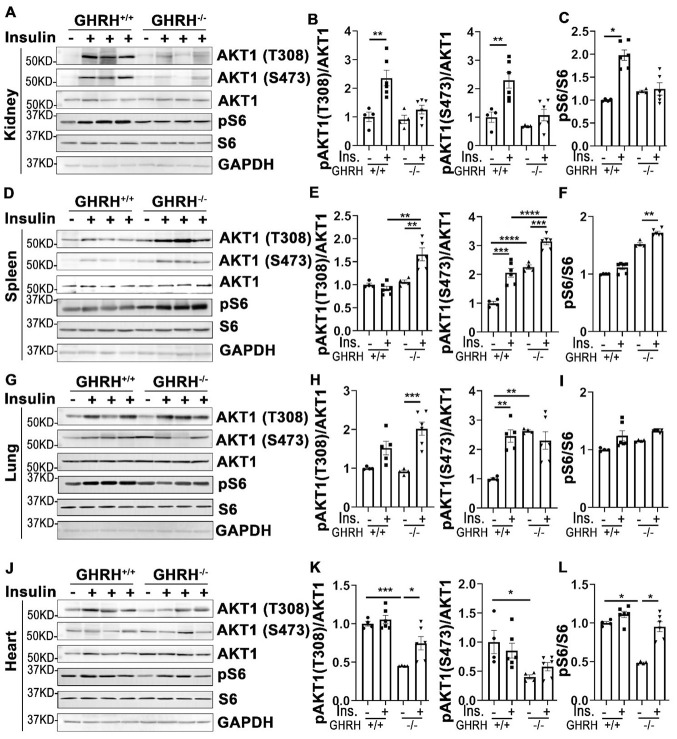
**Organ-specific insulin sensitivity by activation of AKT/S6 in GHRH^-/-^ mice.** (**A**–**C**) Activation of AKT and S6 in kidney. (**D**–**F**) Activation of AKT and S6 in spleen. (**G**–**I**) Activation of AKT and S6 in lung. (**J**–**L**) Activation of AKT and S6 in heart. The mice were fasted for 4 hours before i.p. injection with porcine insulin (1 IU/kg of body weight). Tissues were collected 20 min after following insulin i.p. injection. All data (means ± sem) were expressed as fold change compared to vehicle treated WT controls (defined as 1.0) (n=4 for WT group; n=6 for GHRH^-/-^ mice). Statistical analysis was performed by unpaired Student’s t-test, * *P* < 0.05, ** *P* < 0.01, *** *P* < 0.001 and **** *P* < 0.0001.

In addition to its actions in metabolically active tissues, insulin also acts as a biological mediator to affect the functions of other tissues like kidney, spleen, lung and heart [35]. Thus, we assessed insulin-stimulated response in these tissues. As shown in Figure 4, insulin stimulated phosphorylation of AKT1 on Thr-308 and Ser-473 sites were increased only in kidney of WT controls, but not in GHRH^-/-^ mice (Figure 4A and 4B). A similar pattern was observed in the activation of S6 (Figure 4C).

In spleen, we found that insulin led to a modest activation of AKT1 at both phosphorylated sites and S6 in GHRH^-/-^ mice, whereas it failed to activate AKT1/S6 cascade in the same magnitude in WT controls (Figure 4D–4F).

In lung, basal expression of pAKT1 at Ser-473 site was higher in GHRH^-/-^ mice compared to WT mice, while no difference was observed for the AKT1 on Thr-308 site at the baseline (Figure 4G, 4H, 4J, 4K). Insulin increased phosphorylation of pAKT1 at Thr-308 site in the GHRH^-/-^ mice to similar level as WT controls (Figure 4G and 4H). However, insulin failed to stimulate the phosphorylation of AKT1 (Ser-473) and S6 in GHRH^-/-^ mice (Figure 4G–4I).

Despite the lower basal expression levels of pAKT and pS6 in cardiac tissues, insulin-stimulated phosphorylation of these kinases was significantly higher in GHRH^-/-^ mice, but not in WT mice (Figure 4J–4L).

### Insulin-stimulated activation of ERK signaling in various tissues of GHRH^-/-^ mice

**Figure 5:**
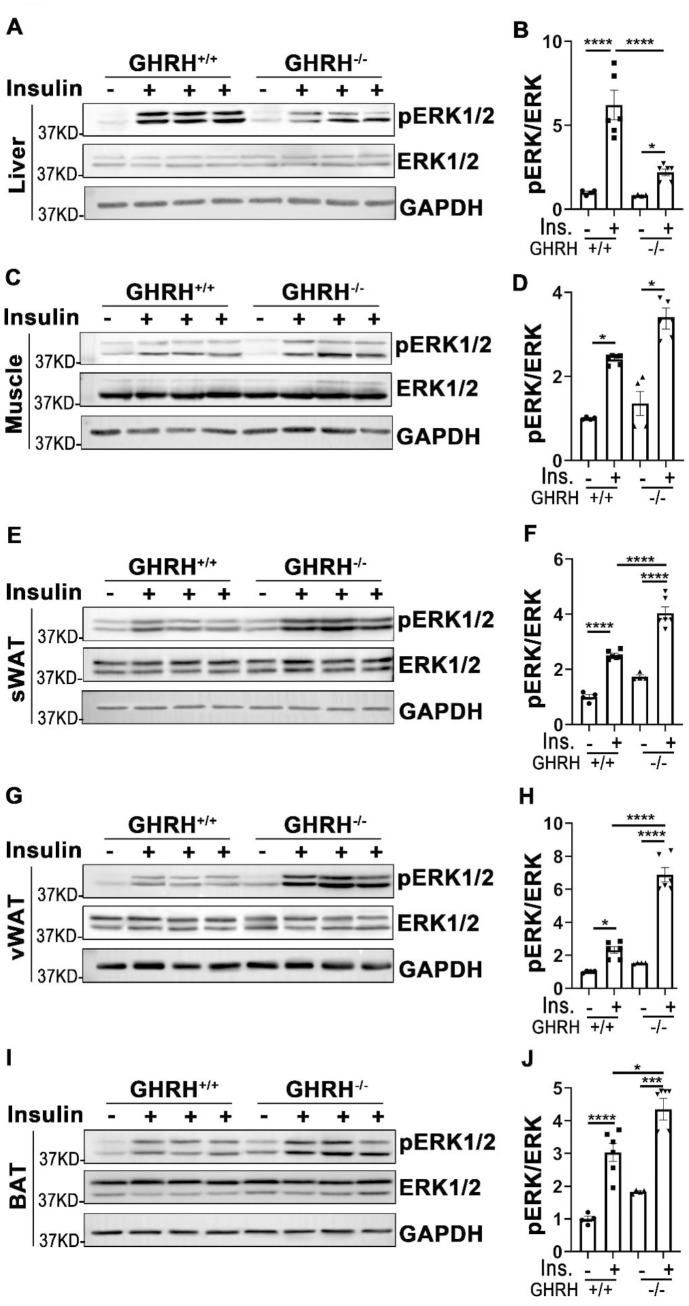
**Analysis of insulin-induced activation of ERK1/2 in metabolically active tissues of GHRH^-/-^ mice.** (**A**, **B**) Activation of ERK1/2 in liver. (**C**, **D**) Activation of ERK1/2 in skeletal muscle. (**E**, **F**) Activation of ERK1/2 in subcutaneous white adipose tissue. (**G**, **H**) Activation of ERK1/2 in visceral white adipose tissue. (**I**, **J**) Activation of ERK1/2 in interscapular brown adipose tissue. The 4 hours fasted mice were injected i.p. with porcine insulin (1 IU/kg of body weight). After 20 min, tissues were collected to perform western blots. All data (means ± sem) were expressed as fold change compared to vehicle treated WT controls (defined as 1.0) (n=4 for WT group; n=6 for GHRH^-/-^ mice). Statistical analysis was performed by unpaired Student’s t-test, * *P* < 0.05, ** *P* < 0.01, *** *P* < 0.001 and **** *P* < 0.0001.

**Figure 6:**
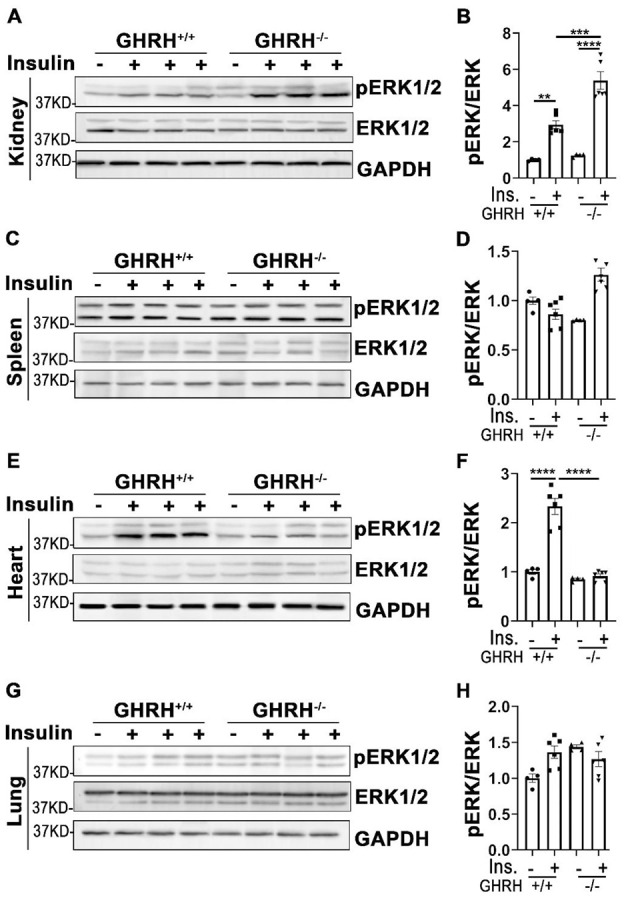
**Tissue-specific phosphorylation of ERK1/2 by insulin stimulation in GHRH^-/-^ mice.** (**A**, **B**) Activation of ERK1/2 in kidney. (**C**, **D**) Activation of ERK1/2 in spleen. (**E**, **F**) Activation of ERK1/2 in heart. (**G**, **H**) Activation of ERK1/2 in lung. The 4 hours fasted mice were injected i.p. with porcine insulin (1 IU/kg of body weight). After 20 min, tissues were collected to perform western blots. All data (means ± sem) were expressed as fold change compared to vehicle treated WT controls (defined as 1.0) (n=4 for WT group; n=6 for GHRH^-/-^ mice). Statistical analysis was performed by unpaired Student’s t-test, ** *P* < 0.01, *** *P* < 0.001 and **** *P* < 0.0001.

In addition to its functions in PI3K/AKT-mediated metabolic pathway, insulin exerts its mitogenic effects via MAPK-ERK cascade [23]. As shown in Figure 5, phosphorylation of ERK1/2 was increased in liver, skeletal muscle, WAT, BAT and kidney of GHRH^-/-^ and WT mice with acute insulin treatment. In GHRH^-/-^ mice, insulin-stimulated phosphorylation of ERK1/2 was significantly higher in skeletal muscle, WAT, BAT and kidney (Figure 5C–5J and Figure 6A, 6B), while lower in liver and heart (Figure 5A, 5B and Figure 6C, 6D) compared to WT mice. Spleen and lung failed to respond to insulin stimulation in both genotypes (Figure 6C, 6D, 6G and 6H). These data indicate that GH deficiency further enhanced insulin sensitivity in skeletal muscle, WAT, BAT and kidney via activated ERK1/2 cascade.

### Attenuated insulin sensitivity in the brain of GHRH^*-/-*^ mice

**Figure 7:**
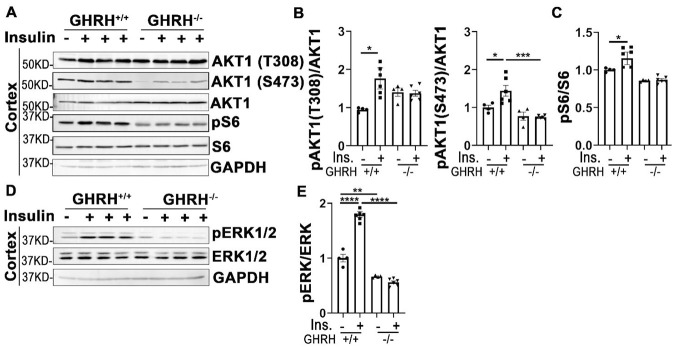
**Cortex attenuated insulin sensitivity in GHRH^-/-^ mice.** (**A**–**C**) Activation of AKT and S6. (**D**, **E**) Activation of ERK1/2. The 4 hours fasted mice were injected i.p. with porcine insulin (1 IU/kg of body weight). After 20 min, tissues were collected to perform western blots. All data (means ± sem) were expressed as fold change compared to vehicle treated WT controls (defined as 1.0) (n=4 for WT group; n=6 for GHRH^-/-^ mice). Statistical analysis was performed by unpaired Student’s t-test, * *P* < 0.05, ** *P* < 0.01, *** *P* < 0.001 and **** *P* < 0.0001.

Insulin signaling plays critical roles in neuronal growth, repair, and metabolism partially via PI3K-AKT and MAPK-ERK1/2 signaling pathways. Our data in Figure 7A–7C showed that insulin significantly activated AKT1/S6 and ERK1/2 in cerebral cortex of WT controls, but not in GHRH^-/-^ mice. Intriguingly, the basal expression of pERK1/2 in GHRH^-/-^ mice was much lower than WT controls (Figure 7D and 7E). These results indicate a brain-specific manner in response to insulin stimulation in the GHRH^-/-^ mice.

### Increased glycogen synthesis associated with inactivation of GSK3α/β in GHRH^*-/-*^ mice

**Figure 8:**
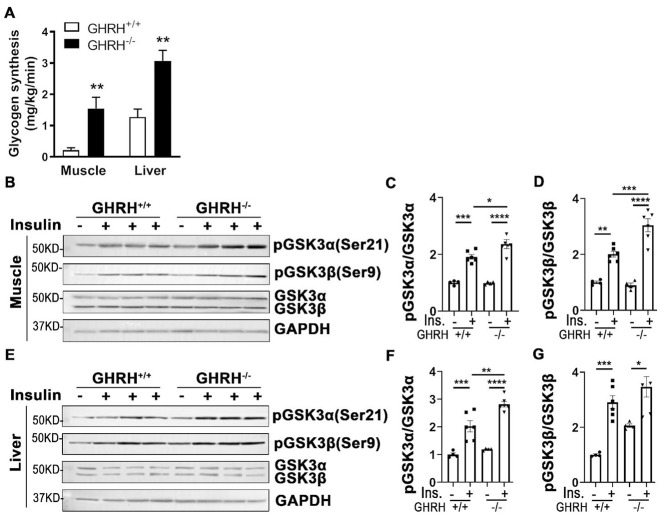
**Increased glycogen synthesis associated with inactivation of GSK3α/β in liver and muscle of GHRH^-/-^ mice.** (**A**) Increased glycogen synthesis in liver and muscle in GHRH^-/-^ mice. All data were represented as mean ± sem (n = 4-5 mice for each genotype). ** P < 0.01 versus vehicle within time points. (**B**–**D**) Activation of GSK3α/β in skeletal muscle. (**E**–**G**) Activation of GSK3α/β in liver. The 4 hours fasted mice were i.p. injected with porcine insulin (1 IU/kg of body weight). After 20 min, tissues were collected to perform western blots. All data were represented as the means ± sem (n=4 for WT group and n=6 for GHRH^-/-^ to detect activation of GKS3α/β). Statistical analysis was performed by unpaired Student’s t-test, * P < 0.05, ** P < 0.01, *** P < 0.001 and **** P < 0.0001.

Insulin clamp data showed that whole body glycogen/lipid synthesis was significantly elevated in GHRH^-/-^ mice (22 mg/kg/min) compared to WT mice (11 mg/kg/min, Figure 2E). Furthermore, glycogen synthesis increased 15-fold in skeletal muscle and 3-fold in liver of GHRH^-/-^ mice compared to WT mice (Figure 8A). Additionally, insulin-stimulated hepatic and skeletal muscular pAKT1 were increased in GHRH^-/-^ mice compared to WT controls (Figure 3A–3F). Glycogen synthase kinase-3 (GSK3) is a substrate of pAKT, thus, we sought to examine whether an increase of glycogen synthesis in skeletal muscle and liver in GHRH^-/-^ mice was associated with insulin-stimulated inactivation of glycogen synthesis kinase (GSK3α/β). Indeed, insulin-induced phosphorylation of GSK3α and GSK3β was significantly higher in liver and skeletal muscle of GHRH^-/-^ mice than WT controls (Figure 8). In contrast, insulin has no effect on GSK3α/β in adipose tissue (Supplementary Figure 1). These results imply that the increased glycogen synthesis in skeletal muscle and liver are associated with AKT-mediated inactivation of GSK3α/β in GHRH^-/-^ mice.

## DISCUSSION

In addition to control of somatic growth, GH acts as a negative regulator of longevity in mammals. Laboratory mouse models with impaired GH signaling, Ames (*Prop-1^df^*) and Snell (*Pit-1^dw^*) dwarf mice [3, 4], GHRKO mice [5], *Ghrhr^lit/lit^* mutation mice [6] and *ghrh^-/-^* mice [7] have prolonged longevity. Diminished secretion rate of GH has been associated with familial human longevity [1]. Intriguingly, the close association between extended longevity and enhanced insulin sensitivity is reported in the context of disrupted GH signaling pathway [36]. GHRH gene functions in somatotroph cell proliferation and GH secretion [37]. GHRH knockout mice (GHRH^-/-^) have significantly decreased expression of GH in the pituitary gland and share the common physiological features with other GH mutant mice, including lower fasting insulin levels and higher insulin sensitivity. We found that GHRH^-/-^ model exhibits lower circulating insulin levels with no difference in glucose basal levels under fasting, indicating that the relationship between insulin and glucose levels is influenced by the insulin sensitivity [38]. Our GHRH^-/-^ model is insulin sensitive, therefore is able to maintain glucose levels similar to WT mice with less circulating insulin. Similar to GHRKO mice, GHRH^-/-^ mice showed a decreased pancreatic islet size [39, 40]. GH promotes pancreatic islet growth and prevents apoptosis in monocytes [41]. Thus, repressed pancreatic islet hyperplasia resulting from GH deficiency might be attributed to either decreased proliferation or increased apoptosis of pancreatic islet cells.

Recent studies of mutant mice with tissue specific reduction of GH signaling have led to an understanding of metabolic alterations of GH action [42]. Information from these new mutant mice brings new insight into the mechanism of local GH actions in the target organs or tissues. Researchers developed muscle-specific GHR mutant models, which present varying metabolic phenotypes. MCK-Cre-GHRKO mouse shares similar characteristics with global GHRKO mice, including improved insulin sensitivity and glucose homeostasis [43]. However, Mef-2c-Cre-GHRKO mouse have decreased insulin sensitivity and glucose tolerance [44]. Hepatic disruption of GHR mouse under the albumin- Cre driver impaired insulin sensitivity and glucose [45]. Adipocyte-specific GHRKO (AdGHRKO) mouse, which was generated using adiponectin Cre, has improved insulin sensitivity and glucose homeostasis [46]. However, aP2-Cre-fat-GHRKO mice show no signs of improved metabolism and have normal glucose homeostasis [47].

Despite more interesting alterations of insulin action and glucose homeostasis reported in various tissue-specific GHR mutations, there are few studies to investigate insulin sensitivity in various tissues. Thus, understanding of insulin sensitivity at the tissue level is critical to elucidate insulin-mediated metabolism and GH/insulin-related improvement of longevity. Taking advantage of long-lived GHRH null mice, our study shows that the liver, skeletal muscle, white and brown adipose tissues are highly sensitive in response to insulin signaling compared to others tissues.

Our hyperinsulinemic-euglycemic clamp data uncovered enhanced insulin sensitivity via elevated glucose infusion rates in GHRH^-/-^ mice compared to WT mice. In the context of GH deficiency, insulin-induced whole-body glucose uptake was increased and glucose production was repressed. These findings are consistent with those in Ames dwarf mice [48]. Insulin-mediated glucose homeostasis in skeletal muscle and adipose tissue is regulated by altered trafficking of GLUT4 from storage vesicles to the plasma membrane [49]. Thus, elevated glucose uptake and glucose transporter activity may result from insulin-modulated GLUT4 trafficking between the plasma membrane and intracellular compartments in GH-deficient mice. Our data indicated an increase in glycogen synthesis in muscle and liver in GHRH^-/-^ mice. At the molecular level, insulin-induced activation of AKT phosphorylated GSK3 leads to inactivation of GSK3 and subsequently dephosphorylation and activation of glycogen synthesis [50]. Indeed, we found that insulin-induced phosphorylation of GSK3α and GSK3β was significantly increased in skeletal muscle and liver of GHRH^-/-^ mice compared to WT controls, which supports the notion that increase of glycogen synthesis is tightly associated with the insulin-stimulated AKT/GSK3 cascade in GH-deficient mice. GH stimulated lipolysis results in free fatty acid flux from adipose tissues to circulation via activation of hormone-sensitive lipase [51]. Our data showed that plasma free fatty acid was lower in GHRH^-/-^ mice compared to WT mice. Taken together, capacity of responding to insulin actions in muscle, liver and brown adipose tissue was significantly enhanced, which, in turn, induces a systemic alteration in glucose and lipid metabolism in the context of disrupted GH signaling pathway.

Insulin and Insulin growth factor-1 (IGF-1) signaling cascades are involved in a series of phosphorylations in PI3K/AKT and MAPK/ERK pathways. Our previous data indicated a significant decrease in expression of IGF-1 in liver of GHRH^-/-^ mice, however, in this study, we used GHRH^-/-^ mice with insulin stimulation for 20 min to investigate tissue-specific insulin sensitivity via PI3K/AKT and MAPK/ERK cascades. Insulin is essential in maintaining glucose homeostasis and physiological metabolism via phosphorylation and recruiting insulin receptor substrate (IRS) proteins, which triggers its intracellular autophosphorylation of their tyrosine residues. This, in turn, leads to the activation of PI3K-AKT cascade to regulate carbohydrate and lipid metabolism. AKT/PKB, a serine/threonine kinase, is a crucial regulator of several cellular processes including cell survival, proliferation and metabolism [51]. Phosphorylation on Thr308 and Ser473 by PDK1 and mTORC2, respectively, is essential for full AKT activation [52]. Our data indicates that GH deficiency promotes hepatic and skeletal muscular insulin sensitivity by activation of AKT. Subcutaneous and visceral white adipose tissues are the main energy reservoirs and secrete several hunger hormones and cytokines that regulate metabolism and insulin resistance [53]. Accumulation of adipose tissue surrounding the intra-abdominal organs, particularly visceral adipose tissue, is associated with obesity, type 2 diabetes, hypertension and cardiovascular disease [54]. Our data indicates that disruption of GH signaling significantly increases insulin sensitivity in subcutaneous and visceral adipose tissues by activation of AKT/S6 cascade, suggesting that disrupted GH signaling significantly improves the insulin sensitivity of white adipose tissue, which might be regulated via increased glucose uptake in white adipose tissue. Brown adipose tissue functions to maintain body temperature and dissipate excess energy to affect body metabolism and regulate insulin sensitivity in mammals and human [55, 56]. GH deficiency boosts insulin sensitivity by activation of AKT/S6 in brown adipose tissue. Insulin-stimulated activation of AKT/S6 suggests that impaired GH signaling attenuates insulin response in kidney and cerebral cortex, while promoting insulin actions in lung. The kidney is a major target of gluconeogenesis and our data indicates that kidney has impaired insulin-mediated activation of AKT, which may explain the modestly impaired IPGTT in GHRH^-/-^ mice.

In parallel with insulin-induced PI3K/AKT signaling pathway in metabolic action, insulin also activates MAPK/ERK cascade in the mitogenic signaling pathway. To investigate the influence of insulin on MAPK-induced mitogen action in different tissues, the phosphorylation level of ERK1/2 was assessed in GH-deficient mice. Insulin stimulated activation of ERK1/2 in liver, skeletal muscle, white and brown adipose tissues, kidney and heart of WT mice. However, disruption of GH signaling attenuated insulin-induced phosphorylation of ERK1/2 in liver and heart. We did not observe insulin-mediated activation of ERK1/2 in spleen and lung of either genotype, which suggests that AKT/S6 might act as major signaling pathway in response to insulin stimulation. Intriguingly, GHRH^-/-^ mice have significantly enhanced capacity of responding to insulin in white/brown adipose tissues and kidney by activation of ERK1/2. MAPK/ERK signaling pathway plays a critical role in brain development and is required for aging-related memory, learning and cognition [57]. In our study insulin failed to promote activation of ERK1/2 in cerebral cortex, this might be due to differences in insulin action time windows between brain and other tissues. Different protocols need to be employed in order to clarify the action of insulin in the central nervous system.

In summary, insulin-mediated metabolic changes are complex actions involving interaction of multiple organs in the long-lived GH mutant mice. Our results suggest improved insulin actions in various tissues via activation of AKT/S6 and ERK1/2 cascades are likely to mediate the prolonged longevity and healthy-aging effects. Future studies are needed to investigate whether perturbations of these pathways in different tissues in normal mice can cause improvements in phenotype similar to GH mutant mice.

## MATERIALS AND METHODS

### Antibody and chemicals

Antibodies (Abs) for phospho-AKT (T308), phospo-AKT (S473), AKT, phospo-ERK1/2 (Thr202/Tyr204), ERK, phospo-GSK3α (Ser21), phospo-GSK3β (Ser9), GSK3α/β and HRP-linked anti-rabbit secondary Ab were purchase from Cell Signaling Technology (Danvers, MA, USA); Ab for GAPDH from EMD Millipore (Billerica, MA, USA); secondary Ab used for detection of GAPDH (Alexa Fluor 488-conjugated) and western blot stripping buffer from Thermo Fisher Scientific (Waltham, MA, USA). Glucose and porcine insulin for glucose tolerance test and insulin tolerance test from Millipore Sigma (Burlington, MA, USA). Ultra-sensitive mouse insulin ELISA kit (Crystal Chem, IL, USA).

### Experimental model and mice genotyping

The GHRH^-/-^ mice were generated in our lab using CRISPR/Cas9 technology and bred on a mixed genetic background to avoid any possible strain-dependent phenotypes [31]. In brief, GHRH^-/-^ mice have a 291 base pairs deletion that eliminates the splice donor site at exon 2, intron 2-3 and a large part of Exon 3 (77 base pairs out of 102 base pairs), showed successful generation a ‘clean’ model for GHRH^-/-^ mice. PCRs were set up using the oligonucleotide primers; 5’-CTTGCTTCTCTCACACTTGC-3’ (forward) and 5’-TTAAAGGGTCGGAGCAGTAG-3’ (reverse). GHRH^-/-^ and WT cage-mate/littermate controls (GHRH^+/+^) were obtained from heterozygous GHRH^+/-^ mating cages. Here, the female 6-7 month-old GHRH^-/-^ mice were not used for our experiments due to their small body size is restricted to measure by hyperinsulinemic-euglycemic clamp. Therefore, the male 6-7 month-old mice were carried out all experiments.

Mice were housed in the Association for Assessment and Accreditation of Laboratory Animal Care–accredited Animal Resources Program facility at the University of Alabama at Birmingham, in accordance with procedures of the Animal Welfare Act and the 1989 amendments to the Act, and all studies followed protocols approved by the University of Alabama at Birmingham Institutional Animal Care and Use Committee. The mice were maintained on a 12 hours light-dark cycle with ad libitum access to food and water.

### Dual-energy X-ray absorptiometry (DXA)

The mice were scanned using the GE Lunar PIXImus DXA with software version 1.45. The mice were anesthetized with the mixture of Isoflurane (3 %) and oxygen (500 ml/min), and then placed in a prostrate position on the DXA imaging plate to scan. For all scans, the head was excluded from the analysis and the data obtained included lean mass and fat mass.

### Glucose and insulin tolerance tests

The mice fasting for 16 hours were intraperitoneally (i.p.) injected glucose with 1 g/ kg of body weight for glucose tolerance test. Blood glucose levels were measured at 0, 5, 15, 30, 60, 90 and 120 min with a PRESTO glucometer (Salem, NH, USA). For insulin tolerance test, 4 hours-fasted mice were i.p. injected porcine insulin with 1IU / kg of body weight. Blood glucose levels were measured at 0, 5, 15, 30, 60, 90 and 120 min. The data of GTT and ITT were present as absolute value.

### Measurement of glucose, plasma insulin and HOMA-IR

Glucose concentration was measured by a PRESTO glucometer in mice with 4 hours fasting. Blood plasma was extracted by centrifugation at 4^o^C for 20 min at 2,000 x g in 4 hours fasted mice. The measurement of plasma insulin was performed with ultra-sensitivity mouse insulin ELISA kit according to the manufacturer’s instructions. Homeostasis model for assessment of insulin resistance (HOMA-IR) was calculated by glucose (ng/dL) X insulin (uM/ml) / 22.5.

### Hematoxylin-eosin staining and analysis of pancreatic islets

Fresh pancreas tissues were collected in GHRH^-/-^ and GHRH^+/+^ mice, following tissues were fixed in 4 % PFA for 16 hours and embedding in paraffin for histological analysis. Different sections (5 μm thick) were deparaffinated and rehydrated to be stained with Hematoxylin-eosin. Images was observed by microscope (OLYMPUS, CKX41).

Images of islets in 5 consecutive cross-sections, including representative sections of the head, body and tail in the pancreas of each mouse, were used for quantitative evaluation. Pancreatic islet area was measured using ImageJ software and was calculated by counting all islets in each section from same the region of pancreas for each genotype. Total 50 pancreatic islets from 5 mice for each genotype were quantitated. Relative ratio of pancreas mass was analyzed by each pancreas mass divided by its body weight.

### Analysis of Hyperinsulinemic-euglycemic clamp

Hyperinsulinemic-euglycemic clamp was conducted by the UAB Small Animal Glycemic Clamp Core as previously described [58]. Briefly, catheters were implanted in chow-fed mice. Four to six days post-op, mice were fasted 5 hours and insulin was infused through the venous catheter. Insulin infusion was primed with a 25 mU bolus at t=0 and then continuous infusion (2.5 mU/kg/min, diluted in saline) was continued for the duration of the clamp. Euglycemia (120 mg/dL) was maintained by adjusting the infusion rate of a 20 % glucose solution. [3-^3^H]-Glucose (Perkin Elmer, Boston, MA) and [1-^14^C]-2 deoxy-D-glucose (MP Biomedicals, Santa Ana, CA) tracers were included to measure glucose turnover and uptake, respectively, as previously described [58]. Blood samples (100 μl) were taken at -120, -60, -30, -5, 90, 100, 110, and 120 min for the assessment of glucose, insulin, and glucose specific activity (SA) in plasma. Following the clamp, mice were euthanized and tissues (gastrocnemius, soleus, extensor digitorum longus (EDL), quadriceps, liver, gonadal white adipose tissue (WAT), and inter-scapular brown adipose tissue (BAT) were snap frozen in liquid nitrogen.

The rate of whole body glycogen and lipid synthesis rates were calculated from plasma samples obtained during the final 40 min of the clamp (t = 80-120min), as previously reported [59]. The tissue-specific glycogen synthesis rate was calculated based upon the radioactivity from [^3^H]-glucose incorporated into glycogen that was recovered in liver or muscles lysates. This isolation was accomplished by incubating tissues in 30 % KOH at 80 ^o^C followed by ethanol-precipitation (-20 ^o^C Celsius 2 hours followed by centrifugation 2,000 rpm for 15 min). The recovered glycogen pellet contains [^3^H]-glucose and measured radioactivity was used for total and synthesis calculations as previously reported [60].

As previously reported [59], data for whole body glucose update and hepatic glucose production represent the mean values during the last 40 min. Whole body glucose disposal was calculated by adding the rate of residual hepatic glucose production during the last 40 min of each insulin clamp to the glucose infusion rate during the same 40 min time period.

### Protein extraction and Western blot

The GHRH^-/-^ and GHRH^+/+^ mice were i.p. injected with insulin (1 IU / kg of body weight) or saline (as vehicle control). After 20 min, the mice were euthanized to collect tissues for the western blots. Organ-specific protein was lysed on ice in RIPA buffer [150 mM NaCl, 1.0% NP-40, 0.1% SDS (sodium dodecyl sulphate), 50 mM Tris-HCl (pH 8.0) and protease inhibitors]. After 20 min of centrifugation at 14,800 rpm at 4 ^o^C. The supernatant was collected and added Laemmli sample buffer [61]. Samples were boiled at 100 ^o^C for 5 min. Western blots were quantified with GeneTools from SYNGENE according to the manufacturer’s instruction.

### Quantification of AKT and ERK1/2 activation

Tissue-specific lysates were collected and used for western blots. Western blots were quantified using GeneTools from SYNGENE according to the manufacturer’s instructions. Abs for pAKT (T308), pAKT (S473) and AKT were used to test AKT activation. Activation of AKT was calculated by dividing pAKT (T308) by AKT and pAKT (S473) by AKT, respectively. Abs for pERK1/2 and ERK were used for detection of ERK activation. Activation of ERK1/2 was calculated by dividing pERK1/2 by ERK1/2. To assess the change of activated AKT and ERK1/2, the relative fold change of activation was determined by all groups versus vehicle treated WT (defined as 1.0).

### Statistical analysis

Statistical analyses were performed with Prism software (GraphPad, La Jolla, CA, USA). All values were presented as means ± sem. Unpaired student’s t-test was performed with *P* < 0.05 considered significant.

## Supplementary Material

undefined
