# Supplementary material for: Insulin sensitivity in long-lived growth hormone-releasing hormone knockout mice
Source: Aging (Albany NY). 2020 Jul 8;12(18):18033–51. doi: 10.18632/aging.103588 (PMC7585079; doi:10.18632/aging.103588)
Supplement: undefined [file aging-12-103588-s001..pdf]

SUPPLEMENTARY FIGURE

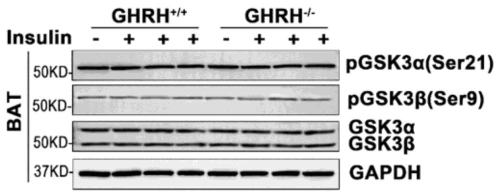

Supplementaru Figure 1. Activation of GSK3α/β in adipose tissue of GHRH$^{-/-}$ mice. The 4 hours fasted mice were injected i.p. with porcine insulin (1 IU/kg of body weight). After 20 min, tissues were collected to perform western blots. n=4 for WT group and n=6 for GHRH$^{-/-}$ mice.
